# Distinct DNA Methylation Profiles in Ovarian Tumors: Opportunities for Novel Biomarkers

**DOI:** 10.3390/ijms19061559

**Published:** 2018-05-24

**Authors:** Lorena Losi, Sergio Fonda, Sara Saponaro, Sonia T. Chelbi, Cesare Lancellotti, Gaia Gozzi, Loredana Alberti, Luca Fabbiani, Laura Botticelli, Jean Benhattar

**Affiliations:** 1 Department of Life Sciences, University of Modena and Reggio Emilia, 41124 Modena, Italy; sergio.fonda@unimore.it (S.F.); sara.saponaro@gmail.com (S.S.); cesarelancellotti@libero.it (C.L.); gaia.gozzi87@gmail.com (G.G.); 2 Unit of Pathology, Azienda Ospedaliero-Universitaria Policlinico, 41124 Modena, Italy; luca.fabbiani@unimore.it (L.F.); botticelli.laura@policlinico.mo.it (L.B.); 3 Institute of Pathology, Lausanne University Hospital, 1011 Lausanne, Switzerland; soniachelbi@msn.com (S.T.C.); alberti.loredana@gmail.com (L.A.); jean.benhattar@gmail.com (J.B.); 4 Aurigen, Centre de Génétique et Pathologie, 1004 Lausanne, Switzerland

**Keywords:** ovarian carcinoma, DNA methylation profiling, Wnt pathway, DNA damage repair system, *TERT*, *MGMT*, *BRCA1*, *MLH1*, *OSMR*, *ESR1*, *FOXL2*

## Abstract

Aberrant methylation of multiple promoter CpG islands could be related to the biology of ovarian tumors and its determination could help to improve treatment strategies. DNA methylation profiling was performed using the Methylation Ligation-dependent Macroarray (MLM), an array-based analysis. Promoter regions of 41 genes were analyzed in 102 ovarian tumors and 17 normal ovarian samples. An average of 29% of hypermethylated promoter genes was observed in normal ovarian tissues. This percentage increased slightly in serous, endometrioid, and mucinous carcinomas (32%, 34%, and 45%, respectively), but decreased in germ cell tumors (20%). Ovarian tumors had methylation profiles that were more heterogeneous than other epithelial cancers. Unsupervised hierarchical clustering identified four groups that are very close to the histological subtypes of ovarian tumors. Aberrant methylation of three genes (*BRCA1*, *MGMT*, and *MLH1*), playing important roles in the different DNA repair mechanisms, were dependent on the tumor subtype and represent powerful biomarkers for precision therapy. Furthermore, a promising relationship between hypermethylation of *MGMT*, *OSMR*, *ESR1*, and *FOXL2* and overall survival was observed. Our study of DNA methylation profiling indicates that the different histotypes of ovarian cancer should be treated as separate diseases both clinically and in research for the development of targeted therapies.

## 1. Introduction

Ovarian cancer is the sixth most common cancer in women worldwide but has the highest mortality rate among gynecologic cancers. More than 90% of ovarian cancers are malignant surface epithelial tumors (carcinomas) and arise from relatively pluripotent cells of the surface epithelium, the coelomic epithelium, or “modified mesothelium” [1,2]. There is now compelling evidence that some carcinomas can also originate from the fallopian tubes and the endometriosis [3]. Ovarian carcinoma is an extremely aggressive disease and only a few cancers are diagnosed at early stages due to the absence of symptoms. In the majority of patients, the disease was characterized by rapid spread into the pelvis and the peritoneal cavity with poor prognosis. Despite the combined surgery and chemotherapy, recurrences are frequent with a low five-year survival rate [4,5].

Ovarian carcinoma is a heterogeneous disease with differences in tumor morphology, clinical evolution, and genetics. The main histological subtypes are serous, mucinous, and endometrioid for their resemblance to the tissues of the female genital tract.

However, some issues about ovarian cancers are still unclear: the origin of most of the ovarian carcinomas is controversial, the rate of relapse is surprisingly high, and its cause is unknown. Therefore, there is an urgent need to better understand the biology of the ovarian cancer in order to improve the diagnosis and to adapt the treatment according to the characteristics of the tumor.

To date, several oncogenes or putative tumor suppressor genes have been implicated in ovarian carcinogenesis [6]. The most commonly activated oncogenes by point mutation are *KRAS*, *BRAF*, and *PIK3CA*. Among the tumor suppressor genes, *TP53, PTEN, BRCA1*, and *BRCA2* are frequently lost or inactivated in epithelial ovarian cancer (EOC). Moreover, several signaling pathways are commonly activated in ovarian carcinomas, including the EGFR, the Ras/MAPK, the PI3K/AKT, the IL6/STAT3, the NF-κB, and the hedgehog pathways [7,8].

Epigenetic mechanisms also contribute to the inactivation or downregulation of tumor suppressor genes. Several tumor suppressor genes, including *CDH1*, *CDKN2A*, *DAK1*, *DLEC1*, *MLH1*, and *RASSF1A*, were found to be downregulated by promoter methylation in ovarian cancer [9]. Furthermore, *BRCA1* (breast cancer type 1 susceptibility gene) was found to be inactivated by promoter methylation as well as by mutation and loss of heterozigosity (LOH) [10,11].

DNA methylation profiles among different types of ovarian cancer indicate that significantly fewer hypermethylated genes are present in high-grade serous carcinomas than in low-grade serous carcinoma and borderline tumors [12]. Various DNA hypermethylation patterns were also identified in different ovarian cancer subtypes, thus suggesting that the cancer origin may involve different biology through different epigenetic mechanisms [13].

In this paper, we analyze the DNA methylation status of a large subset of genes to evaluate if epigenetic alterations can be related to the biological onset of these neoplasias. These putative cancer biomarkers may help to identify relevant biological aspects of ovarian cancer. We used a new approach, Methylation Ligation-dependent Macroarray (MLM) (recently published by Guilleret et al. [14]), to allow the analysis to be more rapid, specific, and efficient, thereby allowing screening of many promoter genes in many samples at the same time.

## 2. Results

**Figure 1:**
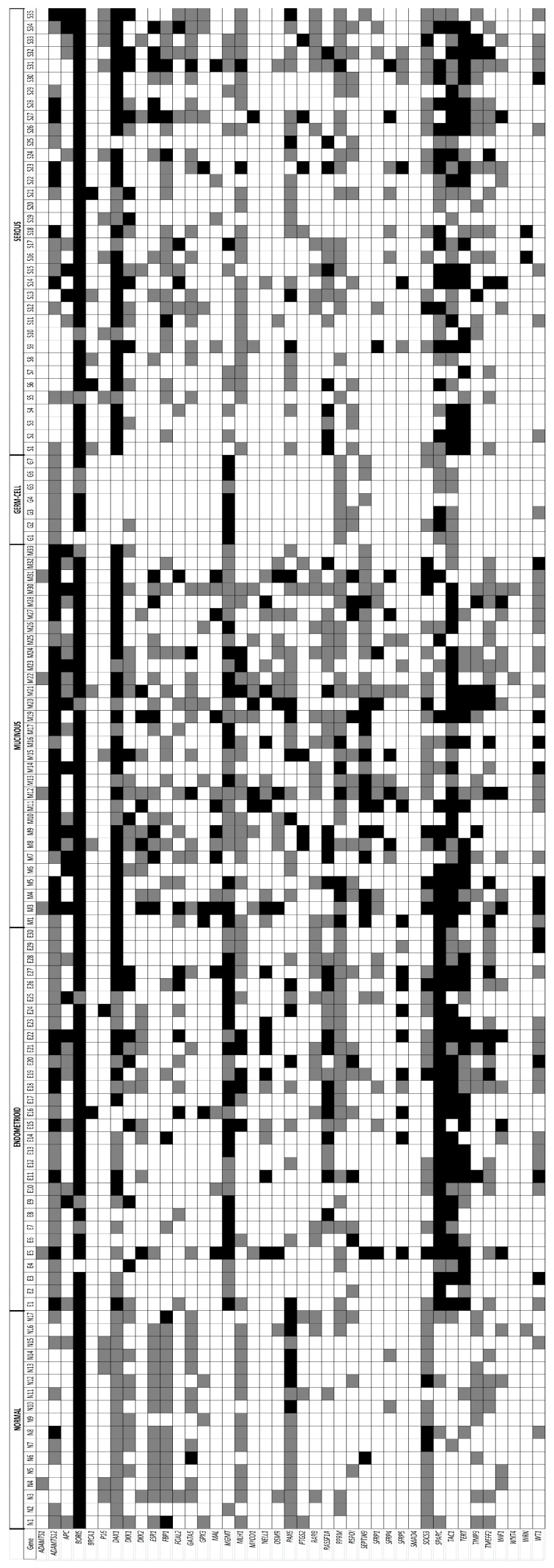
Methylation profiling by MLM in ovarian tumors. Methylation profiling of 41 gene promoters was performed in 17 normal ovarian tissues, and in 102 microdissected ovarian tumor tissues. Each row represents a gene and each column a tissue sample. Black boxes indicate high methylation (β-value > 0.7), grey boxes intermediate or partial methylation (β-value ≥ 0.3 and ≤ 0.7) and white boxes low methylation (β-value < 0.3).

The analysis of DNA methylation profiling of 41 promoter regions using the new MLM technology [14] was performed on 102 ovarian tumors and 17 normal control tissues (Figure 1). The level of methylation was considered low for β-value < 0.3 (white color), partial or intermediate for β-value ≥ 0.3 and ≤ 0.7 (grey color), and high for β-value > 0.7 (black color). A promoter region was considered hypomethylated when the β-value was less than 0.3. This value was chosen because our microdissection guaranteed a percentage of tumor cells greater than 70% and thus a methylation up to 30% could originate from the non-tumor cells. The average number of hypermethylated genes varied depending on the histologic tumor subtypes. For the 17 normal control cases, an average of 29% (11.8/41 genes) of hypermethylated genes was observed. This average percentage increased slightly in the serous (32%; 13.1/41 genes) and endometrioid (34%; 14.1/41 genes) carcinomas, and more significantly in the mucinous (45%; 18.4/41 genes) carcinomas. On the other hand, a decrease in the number of hypermethylated genes was observed in the germ cell tumors (20%; 8.1/41 genes).

Manual microdissection has been performed on formalin-fixed, paraffin-embedded (FFPE) tissues to reach a minimum of 70% of tumor cells. Therefore, a hypo- or a hyper-methylation should be observed for most of the promoter genes in the tumor tissues. However, for almost all the tumors and for most of hypermethylated genes, an intermediate or partial level of methylation (β-value ≥ 0.3 and ≤ 0.7) was noticed (grey color in Figure 1), which could indicate a high level of intratumor heterogeneity in ovarian tumors.

**Figure 2:**
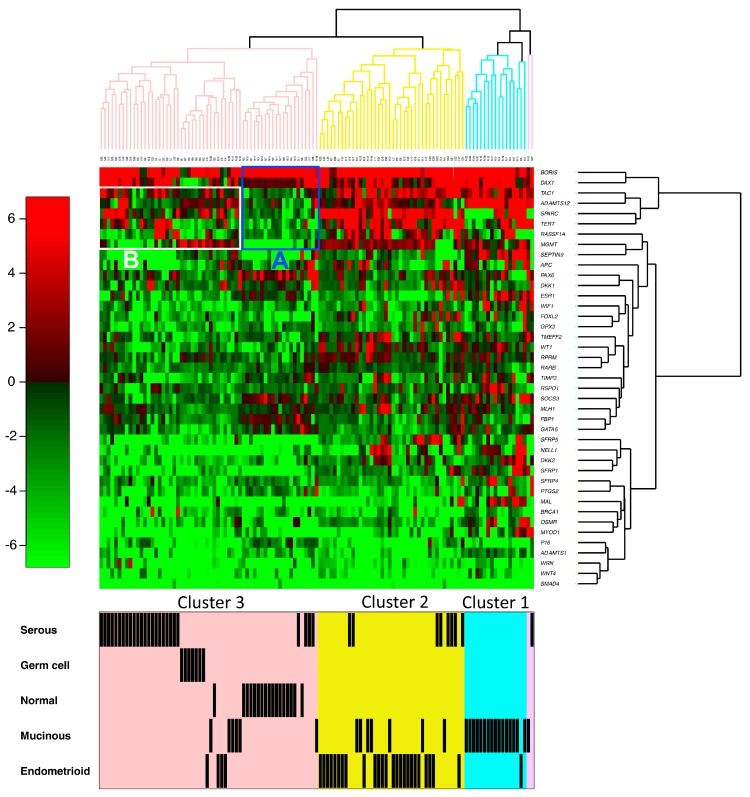
Hierarchical clustering of 41 differentially methylated gene promoters in 102 ovarian tumors and 17 normal ovarian tissues. The methylation levels (M-values) vary from 0 (0% methylation, green) to 1 (100% methylation, red). Cluster 1 (cyan); Cluster 2 (yellow); and Cluster 3 (pink, with groups A and B).

Unsupervised hierarchical clustering identified mainly three clusters of tumors (Figure 2).

The Cluster 1 corresponds to the tumors with the greatest number of hypermethylated genes. All but one of the tumors of this group were mucinous carcinomas. Moreover, more than half of the mucinous carcinomas (53%; 16/30 samples) were contained in this cluster.

The Cluster 2 included almost all the endometrioid carcinomas (83%; 25/30). In addition, few serous and mucinous carcinomas (seven cases for each group) were also present in this cluster.

The Cluster 3 corresponds to the tumors with the lowest number of hypermethylated genes. These cluster included all normal ovarian tissues, all germ cell tumors, and the majority of serous carcinomas (74%; 26/35 samples). In addition, few mucinous and endometrioid carcinomas (6 and 4 cases, respectively) were also present in this cluster. The Cluster 3 can be divided in two groups, 3A and 3B. The Group 3A contained all but one of the non-tumoral tissues. The Group 3B contained all the germ cell tumors and the majority of the serous carcinomas (63%; 22/35 samples). The presence of normal tissues and germ cell tumors could alter the clustering of epithelial tumors. To exclude this possibility, a clustering including only the epithelial tumors was performed. Three clusters were once more obtained, with the majority of mucinous carcinomas in the first one, the majority of endometrioid carcinomas in the second one, and more than two-thirds of serous carcinomas in the third cluster (Figure S1).

Some promoter genes—*ADAMTS12*, *MGMT*, *SPARC*, *TAC1*, and *TERT*—were hypomethylated in most normal tissues (Figure 1 and Group A in Figure 2) and were hypermethylated in a majority of tumors. However, this change in methylation level was not uniform and, for some genes, it will be different according to the tumor type. *ADAMTS12* and *MGMT* were hypermethylated in almost all of the endometrioid, mucinous, and germ cell tumors. On the other hand, in serous carcinomas, only 51% and 31% of the tumors were found hypermethylated for *ADAMTS12* and *MGMT*, respectively. For the *TERT* gene, the number of hypermethylated tumors was dependent on the histological type. The endometrioid carcinomas were very often *TERT* hypermethylated whereas the opposite occurred in germ cell tumors, respectively, 83% and 11%. *TERT* methylation was observed in 71% of serous and 50% of mucinous carcinomas.

In normal tissues, several genes—*DAX1*, *ESR1*, *FBP1*, and *SOCS3*—showed an intermediate methylation level in more than three-quarters of samples. The case of *DAX1* is particularly interesting. With the exception of germ cell tumors, where hypomethylation is observed in all but one case, a majority of mucinous and serous carcinomas showed a strong hypermethylation of *DAX1* (black boxes in Figure 1).

There are six genes (*MAL*, *MYOD1*, *OSMR*, *SEPTIN9*, *SFRP1*, and *SFRP4*) for which hypermethylation is observed almost exclusively or preferentially in mucinous carcinomas (Figure 1). Moreover, for each of these genes, at least two-thirds of tumors with hypermethylation were present in cluster 1 (Figure 2). With the exception of *APC*, a hypermethylation of the genes belonging to the Wnt/β-catenin signaling pathway—*DKK2*, *SFRP1*, *SFRP4*, *SFRP5*, and *WIF1*—appeared mainly in the clusters 1 and 2. All these observations are confirmed by the analysis with the Student’s *t* test with unequal variances at *p* < 0.05.

The MLM analysis revealed that some genes—such as *ADAMTS1*, *CDNK2A* (p16), *PTGS2*, *SMAD4*, *WNT4*, and *WRN*—were never or uncommonly found hypermethylated in our series of tumors and normal ovarian tissues.

The survival analysis was possible for 86 of the 95 patients with ovarian carcinoma. For nine cases, two endometrioid, three serous, and four mucinous, the data were not available. The five-year overall survival revealed that the median overall survival times was 47 months for serous carcinomas, 44 months for the mucinous carcinomas, and 56 months for the endometrioid carcinomas. Fifteen patients died of the disease for serous carcinoma (all but three were at stage 3), ten for mucinous carcinoma (stage 3 was observed in four cases, 2 in one, and 1 in five cases), and three for endometrioid carcinoma (one at stage 1, one at stage 2, and one at stage 3). Recurrence was observed in nine cases of serous carcinoma (range 8–48 months), in one mucinous carcinoma (at 13 months), and in one endometrioid carcinoma (at 12 months). Our cohort is not completely representative for EOC due to the relatively low number of serous carcinomas that are the most frequent in the general population and are usually most advanced cancers. The statistical survival analysis showed interesting results. Among all the patients with ovarian carcinomas harboring a methylation of *OSMR* or *ESR1*, β-value > 0.3 and M-value > −1.222, we found more patients dead of the disease than in the cases with a hypomethylation of these genes (*p* < 0.05). Moreover, a statistically significant difference in mean overall-survival between patients with carcinomas harboring high methylation levels of *OSMR* (β value > 0.7 and M-value > 1.222) compared to patients with carcinomas less methylated (12.5 vs. 25.0 months) was observed (*p* < 0.05). For two genes, *RASSF1* and *FOXL2*, the patients who died of the ovarian carcinoma showed a statistically higher mean of methylation compared to the patients who were still alive (M-value of 6.63 vs. 4.16 for *FOXL2* and 6.43 vs. 5.17, for *RASSF1*; *p* < 0.05).

The Kaplan-Meier method (R package “survival”, CRAN) was applied to widen the analysis procedure (used function: survfit).

The overall survival curves were generated analyzing the survival behavior as a function of two methylation levels, under and over a β-level threshold. The Log rank test was used to test the significance of the difference between the two curves (used function: survdiff).

**Figure 3:**
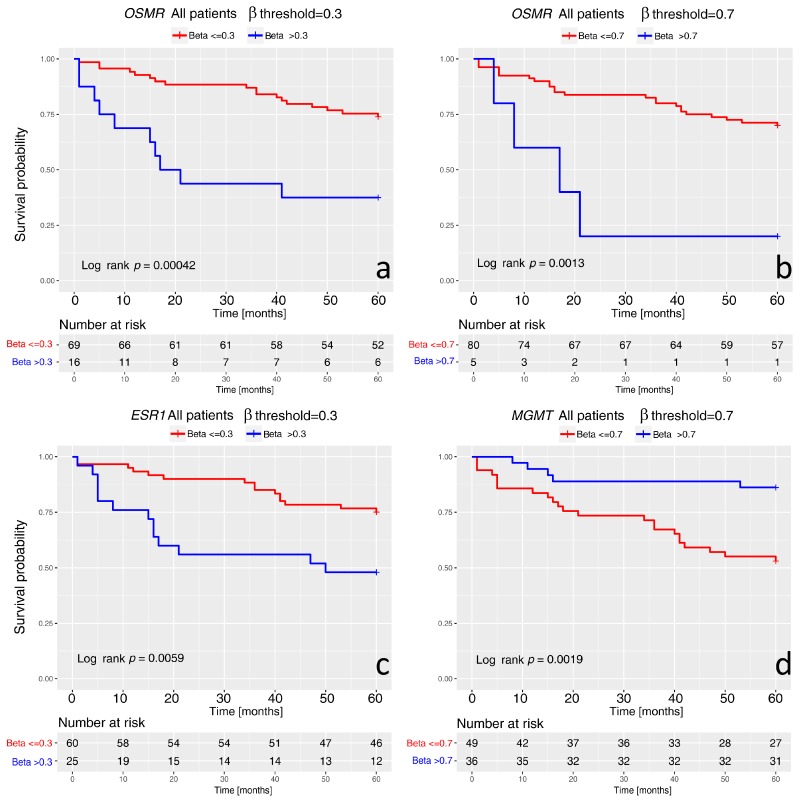
Kaplan-Meier analysis of overall survival of epithelial ovarian cancer patients. The analysis was carried out through the function “survfit” of the R package “survival” (ver. 2.42-3, Available online: https://cran.r-project.org/web/packages/survival/index.html) with the log-log mode setting. Each graph legend reports the β threshold and their colors (red, blue) and the *p* value of the test for the significant difference between the curves. Curves for *OSMR* gene were generated with two threshold levels of β methylation, 0.3 and 0.7 (**a**,**b**), respectively. Curves for *ESR1* gene were generated with threshold 0.3 (**c**). The curves for *MGMT* gene were generated with threshold of 0.7 (**d**). The number of patients in each subgroup is reported in the table at the bottom of each graph. The censored cases are marked by “+”.

The curves for *OSMR* were obtained with two threshold levels, 0.3 and 0.7. In Figure 3a, the curves show the threshold 0.3. Patients with unmethylated carcinoma (β-value < 0.3, blue curve) show a better overall survival (Log rank *p*-value = 0.00042). The aspect of “shorter survival for larger methylation” is reinforced by the results obtained with the threshold of β-value = 0.7. The corresponding survival curves are reported in Figure 3b where the gap between them is much larger if compared to that for lower threshold (Log rank *p*-value = 0.0013).

We had similar results for the curves related to *ESR1*, reported in Figure 3c, where they appear well separated (Log rank *p* value = 0.0059).

**Figure 4:**
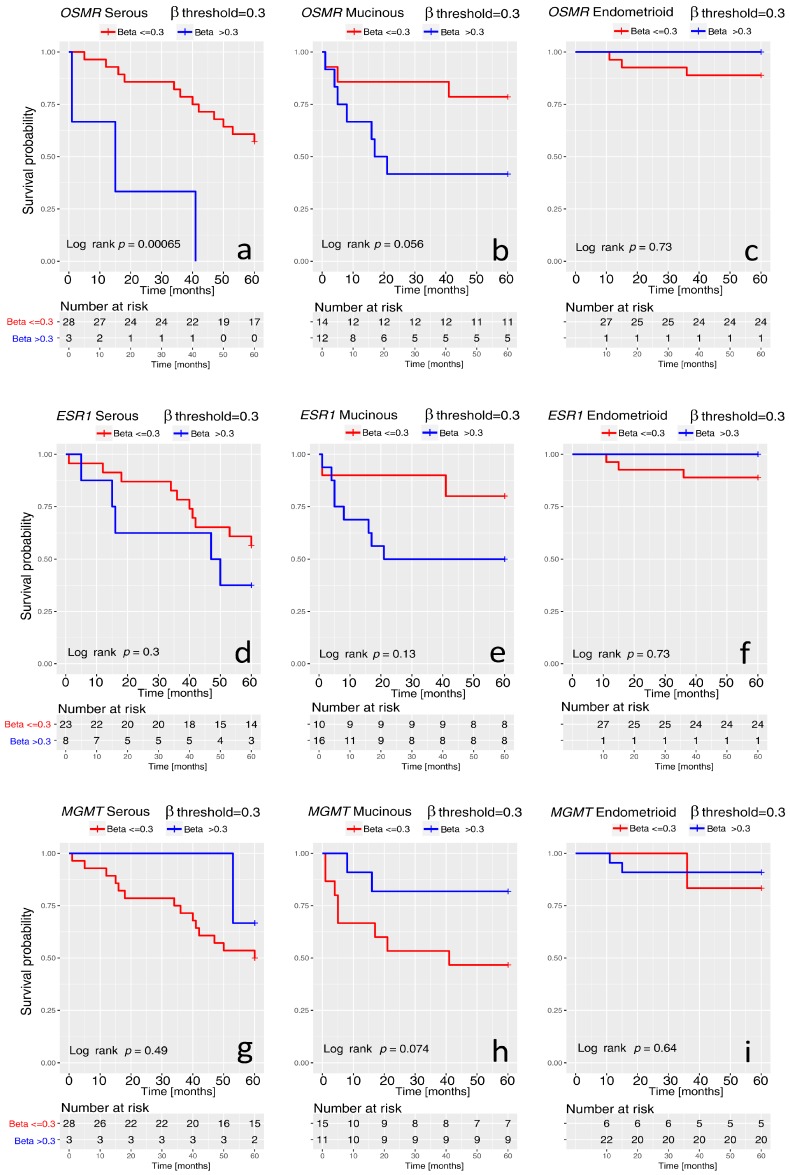
Kaplan-Meier analysis of overall survival of ovarian carcinomas stratified for their histology: S (Serous), M (Mucinous), E (Endometrioid). Curves were generated for *OSMR* (upper panel) (**a**–**c**), *ESR1* (central panel) (**d**–**f**), and *MGMT* (lowest panel) (**g**–**i**). In each graph, the β threshold and their colors (red, blue) and the *p* value are reported. The number of patients in each subgroup is reported in the table at the bottom of each graph. The censored cases are marked by “+”.

An opposite behavior of the overall survival curves was found for *MGMT* gene, probably due to its different association in specific histotypes of ovarian carcinoma. High methylation of *MGMT* seems to favorite a higher survival probability, as shown in Figure 3d, where the reversed blue and red curves look well separated (Log rank *p* value = 0.0019). We improved the survival analysis by stratifying the samples for their histology and comparing the difference of survival curves between the histotypes by Log rank test. The survival curves for *OSMR*, *ESR1*, and *MGMT* showed a similar feature (Figure 4). A link between hypermethylation and survival was observed for serous and mucinous carcinomas only (Figure 4a,b,d,e,g,h). For endometrioid histotype only one patient presented high methylation. *OSMR* and *ESR1* hypermethylation were associated with worse survival, whereas *MGMT* hypermethylation provided better survival. Due to the limited number of samples in the different subgroups, statistical significance was reached only for *OSMR* in serous carcinomas.

## 3. Discussion

Aberrant methylation of multiple promoter CpG islands is a common event in human cancers and is frequently observed in ovarian carcinomas [15]. It has thus been suggested that DNA methylation changes may have implications for ovarian cancer diagnosis, prognosis, and treatment [16,17]. However, it is currently difficult to compare the results obtained from several previous studies for DNA methylation analysis due to the different methods of analysis, the quantity of genes studied, and the histological types investigated. In our study, using a new technique, Methylation Ligation-dependent Macroarray (MLM) (recently published by Guilleret et al. [14]), we focused our analysis on the three major types of epithelial carcinomas and on a large set of genes involved in several pathways, with the aim of better understanding the biological onset of this neoplasia.

There are three major results that can be drawn from this study regarding the methylation profiles of ovarian cancers.

Firstly, the profiles of methylation are quite heterogeneous, but clustering showed, however, that, at the epigenetic level, there are different groups of tumors. The most interesting point is that these groups reclassified by clustering are very close to the histological classifications. This observation has been reported in previous studies [13,18,19,20,21,22,23,24,25,26] in which a small number of cases or a small number of genes have been considered. Our work highlights and confirms the link between methylation and histotypes due to relatively high number of cases analyzed (95 ovarian carcinomas and 7 germ cell tumors) and to the selection of several genes involved in the carcinogenesis and ovarian development. The connection between DNA methylation profiles and histological subtypes is suggestive of various tumorigenic mechanisms and cells of origin and underlines the need to consider the different histological ovarian carcinomas as different diseases. Treatment strategies require a better understanding of the biology of each histological type.

In addition, our study also seems to confirm that none of the histological subtypes analyzed have their origin in the majority of cells that make up the ovary.

Secondly, an intermediate level of methylation (about 50%) was observed for most hypermethylated genes. Normally, either a high or a low level of methylation should have been observed because the proportion of tumor cells in our samples was greater than 70%. This observation had already been made for analyses of methylation on a single gene by our group but also by others [27,28]. However, our study of 41 genes indicates that this phenomenon is quite general. This observation could mean that some intratumoral heterogeneity exists for these various epigenetic alterations. Of course, it is also possible that, for some genes, a methylation of a single allele occurs; in this case, gene expression by the non-methylated allele is possible and therefore this monoallelic methylation does not lead to a stop of expression.

Thirdly, in our series of ovarian tumors, the number of hypermethylated promoter genes is relatively low for tumors that are considered epithelial. This observation particularly involves serous and endometrioid carcinomas. Using the same MLM methodology, the number of hypermethylated genes was found to be significantly higher in esophagus adenocarcinomas [14]. *TERT* (human telomerase reverse transcriptase) transcription, which leads to telomerase activity, is one of the most important factors linked to proliferation, differentiation, and senescence, and it is not found in the majority of somatic cells. On the contrary, in the proliferative cells such as germ cells, stem cells, and in the 80–95% of the tumors, *TERT* is expressed, which leads to telomerase activation [29,30]. Our previous studies on the regulation of the *TERT* gene showed that the promoter of this gene is hypermethylated in 80–90% of epithelial tumors [31,32,33]. As expected, *TERT* was not methylated in our series of non-epithelial germ cell tumors; on the other hand, *TERT* is hypermethylated in 83% of endometrioid carcinomas but in only 71% of serous and 50% of mucinous carcinomas.

An important observation, which could have strong therapeutic implications, is the aberrant promoter methylation of three genes (*BRCA1*, *MGMT*, and *MLH1*) playing important roles in the different DNA repair mechanisms [34]. Unrepaired DNA damage by the DNA damage repair (DDR) system is a major cause of mutagenic lesions. In human cancer, DNA methylation occurs more frequently than gene mutation in DDR genes [35]. As such, DNA methylation could play more important roles in DNA damage repair genes during the development of cancer. Interestingly, aberrant DNA methylation of DDR genes is a powerful biomarker for precision therapy in cancer [35].

Platinum agents and Poly(ADP-ribose) polymerase (PARP) inhibitors, a promising novel therapy, are the most successful anticancer reagents in BRCA-deficient cancer cells [36]. *BRCA1* and *BRCA2* germline mutations are the most frequently responsible for hereditary breast and ovarian cancer.

Besides mutation, hypermethylation of the promoter of *BRCA1*, which is observed in approximately 10–15% of sporadic ovarian carcinomas, and more especially in serous carcinomas, could also predict response to PARP inhibitors [37]. There are actually very few clinical studies on the role of *BRCA1* methylation in the response to PARP inhibitors (PARPi), both in breast and ovarian cancers. Many papers discuss the strong potential of this marker for PARPi response, but clinical studies are still too few. A recent review article [37] discusses the results of clinical trials and the relation between *BRCA1/2* and PARPi in serous ovarian carcinomas. Apparently the long-term response is much more pronounced for mutated tumors than for hypermethylated tumors. Using MLM, *BRCA1* was found hypermethylated in 14% (5/35) of our series of serous tumors.

Defective mismatch repair (MMR) increases mutation rates and leads to microsatellite instability (MSI), resulting in carcinogenesis. In epithelial ovarian cancer, microsatellite instability (MSI) was observed in 14.9% of cases in a series of 834 samples [38]. MSI can be due to germline mutation within at least one of four mismatch repair genes (*MLH1*, *MSH2*, *MSH6*, and *PMS2*), but more likely to hypermethylation of the MLH1 promoter in sporadic cancers, including ovarian cancers, with an occurrence of 37.5% [39]. With the exception of germ cell tumors, hypermethylation of *MLH1* was observed in more than half of our series of ovarian epithelial tumors. This result is in agreement with the few data from the literature, in which there is apparently no correlation between *MLH1* methylation and the histological subtype [40]. However, it may also be noted that normal ovarian tissue also exhibited a partial methylation of *MLH1* in 76% (14/17) of the analyzed cases. Promoter methylation inhibits transcription when both alleles are methylated. It is therefore possible that the observed methylation in some of the tumors comes either from the normal tissue or from the methylation of a single *MLH1* allele in the tumor cells, which in both cases does not lead to an inhibition of *MLH1* in the tumor. In our series, a complete methylation of *MLH1* was observed for 11 cases, where silencing of *MLH1* expression can therefore be strongly suspected. Immunotherapy has shown promising results in various types of cancers, including those with microsatellite instability-high (MSI-H) or MMR-deficiency [41]. Further studies seem necessary to better understand the effect of *MLH1* methylation in ovarian cancers.

Aberrant hypermethylation of *MGMT* (*O*-6-Methylguanine-DNA Methyl Transferase) is particularly interesting. This gene is involved in the repair of DNA damages and hypermethylation is observed in several neoplastic and preneoplastic conditions [35,42] Recently, a meta-analysis that evaluated the samples of 10 ovarian cancer studies showed the importance of *MGMT* methylation in ovarian cancers [26]. Our study confirms the high occurrence of *MGMT* methylation in ovarian cancers. We observed that complete methylation of *MGMT* was present in endometrioid (60%, 18/30), mucinous (33%, 10/30), and germ cell (86%, 6/7) subtypes, but almost absent in serous carcinoma (3%, 1/35). *MGMT* promoter methylation was significantly correlated with pathological types, and it was significantly lower in serous carcinomas than in non-serous carcinomas. The association of *MGMT* methylation with certain histological types was previously observed. This hypermethylation was described as common in most histological subtypes, with the exception of serous carcinoma [43,44], as shown in our study. Our data on survival analyses showed that *MGMT* hypermethylation leads to a higher survival rate. This could be due to its role in DNA repair and thus, a better response to chemotherapy, as observed in glioblastoma [45] but also in advanced ovarian cancer [46]. In our cases, the link between hypermethylation and better survival was found particularly for serous and mucinous histotypes. Further studies conducted on large series of carcinomas, and more importantly with distinction of the different histotypes, are required to determine the precise role of *MGMT* methylation in ovarian tumorigenesis and outcome.

Aberrant activation of the Wnt/β-catenin pathway plays an important role in a wide variety of cancers [47]. This activation occurs throughout genetic or epigenetic alterations of intracellular or extracellular components of this pathway. Wnt activation has been observed in several cancer types, including colorectal carcinoma, pancreatic adenocarcinoma, leukemia, melanoma, but also in ovarian carcinoma [48]. In our series, hypermethylation of the Wnt antagonists (*DKK1*, *DKK2*, *SFRP1*, *SFRP4*, *SFRP5*, and *WIF1*) were observed with a significant prevalence in mucinous and endometrioid carcinomas. Promoter methylation of *SFRP4* and *SFRP5* has been associated with advanced stage of the disease and resistance to chemotherapy [11,49]. Given the critical roles of the Wnt/β-catenin pathway in many cancers, substantial efforts have made to develop therapeutic approaches to target this pathway, which could be useful for treating ovarian cancer.

Hypermethylation of *FOXL2* (Forhead box L2), *ESR1* (Estrogen Receptor 1), and *OSMR* (Oncostatin M receptor) was rather indicative of poorer overall survival and could be used as a biomarker of prognosis. Interestingly, for *OSMR* and *ESR1* the link between hypermethylation and worse survival was especially noted for serous and mucinous histotypes in our study. These data are promising, but detailed studies on a more number of patients are needed. It is interesting to note that these three genes play a role in ovarian cells. The transcription faction *FOXL2* (Forhead box L2) has a key role in all stages of ovarian development and maintenance, and regulates apoptosis, proliferation, and differentiation of follicular cells [50]. *ESR1* is involved in sexual development and *OSMR* (oncostatin M receptor), a cell surface receptor, has a possible role with OSM in growth initiation of primordial follicles [51]. At least for one of these three genes (*ESR1*), an inverse correlation between methylation and survival has previously been observed [52].

In conclusion, our study highlighted the large heterogeneity of DNA methylation profiles in ovarian cancers. Nevertheless, clustering identified four groups significantly associated with the known ovarian cancer histological subtypes. This observation supports the need to treat the different histologies of epithelial ovarian cancer as different diseases, both clinically and in research for the development of targeted therapies. The differentially methylated genes we have identified or confirmed in this study may provide clues into the biological characteristics of the different cancer subtypes. Taking into account the high potential of epigenetic biomarkers, further research may lead to successful new drug targets for the development of subtype-specific therapies for ovarian cancer.

## 4. Materials and Methods

### 4.1. Patients and Tissues

The study was conducted on ovarian tissues obtained from women surgically treated for an ovarian carcinoma without preoperative chemotherapy. The Ethics Committee of Azienda Ospedaliero-Universitaria di Modena (project identification code 167/16) approved the study on 20 July 2016. The samples were collected at the Unit of Pathology of Modena between 1995 and 2005 and included 35 serous carcinomas, 30 mucinous carcinomas, and 30 endometrioid carcinomas. Seven germ cell tumors (six dysgerminoma and one embryonal carcinoma) were added to the study. The patients with mucinous carcinomas were carefully evaluated in search of a primary mucinous carcinoma at another site. We excluded the cases with positive immunostaining for CDX2, CEA, or CK20 (more specific for intestinal origin) or negative for CA125. Table 1 reports the clinical and pathological features of patients with ovarian tumors.

The stage was established according to the International Federation of Gynecology and Obstetrics (FIGO) criteria [53]. The pathological grade was specified by the Silverberg grading system (grades I–III) [54] and for serous tumors in low and high-grade according to the recently proposed two-tier grading system [55].

For control cases, we selected 17 normal ovarian tissues from women without cancer who has surgery for benign gynecological conditions (mean age of 58 years). Tissues were formalin fixed, paraffin embedded, and cut to obtain sections of 3–4 μm. After staining with hematoxylin and eosin, the sections were observed by pathologists in order to confirm the histopathological diagnosis.

### 4.2. Microdissection and DNA Extraction

The sections were deparaffinized, rinsed and stained in 0.1% toluidine blue for 30 s, washed, air dried, and stored before microdissection. This microdissection was made manually using a scalpel blade under microscopic control to remove a majority of the non-neoplastic tissue, such as immune cells or necrosis, in order to enrich the samples to at least 70% of tumor cells. The pathologist (LL) confirmed the accuracy of this procedure before the collection of the enriched tumor samples. DNA was extracted using the Promega Maxwell 16 FFPE Plus LEV DNA Purification Kit (Promega, Madison, WI, USA) following manufacturer’s instructions. DNA quantification was performed using standardized PicoGreen fluorescence assay (Invitrogen, Carlsbad, CA, USA).

### 4.3. Methylation by Methylation Ligation-Dependent Macroarray (MLM)

To analyze many promoter genes in many samples at the same time, a methylation macroarray has been developed. A homemade methylation macroarray, termed Methylation Ligation-dependent Macroarray (MLM), has been generated using promoters known to undergo methylation changes in cancers. The method consists of five steps, as previously reported [14].

The methylation level was expressed by the β-values, which were calculated as follows: β = (intensity value in assay tube/intensity in control tube) divided by the mean β value obtained for the restriction site free targets. Finally, β ≤ 0 were replaced by 0.01 while β ≥ 1 were replaced by 0.99. The level of methylation was considered low for β-value < 0.3, partial or intermediate for β-value ≥ 0.3 and ≤ 0.7, and high for β-value > 0.7.

### 4.4. DNA Methylation Unsupervised Hierarchical Clustering and Statistical Analysis

After data conversion from β-values to M-values in which M= log_2_ {β/(1-β)} , the M-values were first submitted to exploratory analysis of the methylation expression and, after statistical survival analysis, to hierarchical clustering by means of the program R and Bioconductor software and package survival (R Bioconductor package Heatplus v2.18.0 by Alexander Ploner, 2014) [56]. The exploratory analysis was carried out by generating boxplots for every kind of each patient’s condition and ordering them as a function of median M-values.

The clusters associated to heatmap were generated with Euclidean distance between M-values and complete distance between clusters. The cut thresholds for patients and gene clusters were 40.0 and 2.0, respectively.

### 4.5. Genes Investigated

The 41 promoter genes investigated are genes that are known to be able to be regulated by a methylation change of their promoters. Among them, we selected some genes known to be altered by DNA methylation in ovarian cancers (*APC*, *ESR*, *MGMT*, *RASSF1A*, *MLH1*, *TERT*, *WT1*), genes implicated in testis and/or ovarian cells (*BORIS*/*CTCFL*, *DAX1*, *FOXL2*, *RSPOI*, *TMEFF2*), genes involved in the Wnt pathway (*APC*, *DKK1*, *DKK2*, *DKK3*, *SFRP1*, *SFRP4*, *SFRP5*, *WIF1*, *WNT4*), and genes implicated in repair pathways (*BRCA1*, *MGMT*, and *MLH1*).

### 4.6. Survival Analysis

Patients were followed-up according to the standard protocol every 6 months for 5 years. Optimal debulking surgery was received in all but six patients (four serous carcinomas and two mucinous carcinomas). Platinum-based chemotherapy treatment was applied for all patients at advanced stages (3 and 4) and to 24 patients at stage 1C and 2.

Overall survival, defined as the period from surgery to the date of death associated to the disease, and disease-free survival, defined as the time from the date of primary treatment to the date of confirmed recurrence, were evaluated by the investigation of clinical files and pathological reports of patients. The statistical analysis of survival was performed using the Kaplan-Meier method to build the survival probability curves. The analysis was carried out through the functions “surv”, “survfit”, “survdiff”, the log-log mode setting, and “ggsurvplot” from the R packages “survival” and “survminer”, respectively (ver. 2.42-3, Available online: https://cran.r-project.org/web/packages/survival/index.html and ver. 0.4.2, Available online: https://cran.r-project.org/web/packages/survminer/index.html). We used the statistical Log rank test to test the null hypothesis for the difference of survival curves.

## Figures and Tables

**Table 1:** Clinical and pathological features of patients with ovarian tumors.

Neoplasia	Number	Mean Age	Grade	Stage
Serous carcinoma	35	58	Low grade: 5 High grade: 30	I = 11
				II = 4
		Min = 37		III = 19
		Max = 87		IV = 1
Mucinous carcinoma	30	59	I = 15	I = 24
		Min = 30	II = 8	II = 3
		Max = 85	III = 7	III = 3
Endometrioid carcinoma	30	60	I = 14	I = 22
		Min = 36	II = 9	II = 5
		Max = 88	III = 7	III =3
Germ cell tumor	7	25	-	I = 6
		Min = 12		
				II = 1
		Max = 33		

## Supplementary Materials

Supplementary materials can be found at http://www.mdpi.com/1422-0067/19/6/1559/s1.
