# Supplementary figures and images for: Distinct DNA Methylation Profiles in Ovarian Tumors: Opportunities for Novel Biomarkers
Source: Int J Mol Sci. 2018 May 24;19(6):1559. doi: 10.3390/ijms19061559 (PMC6032431; doi:10.3390/ijms19061559)

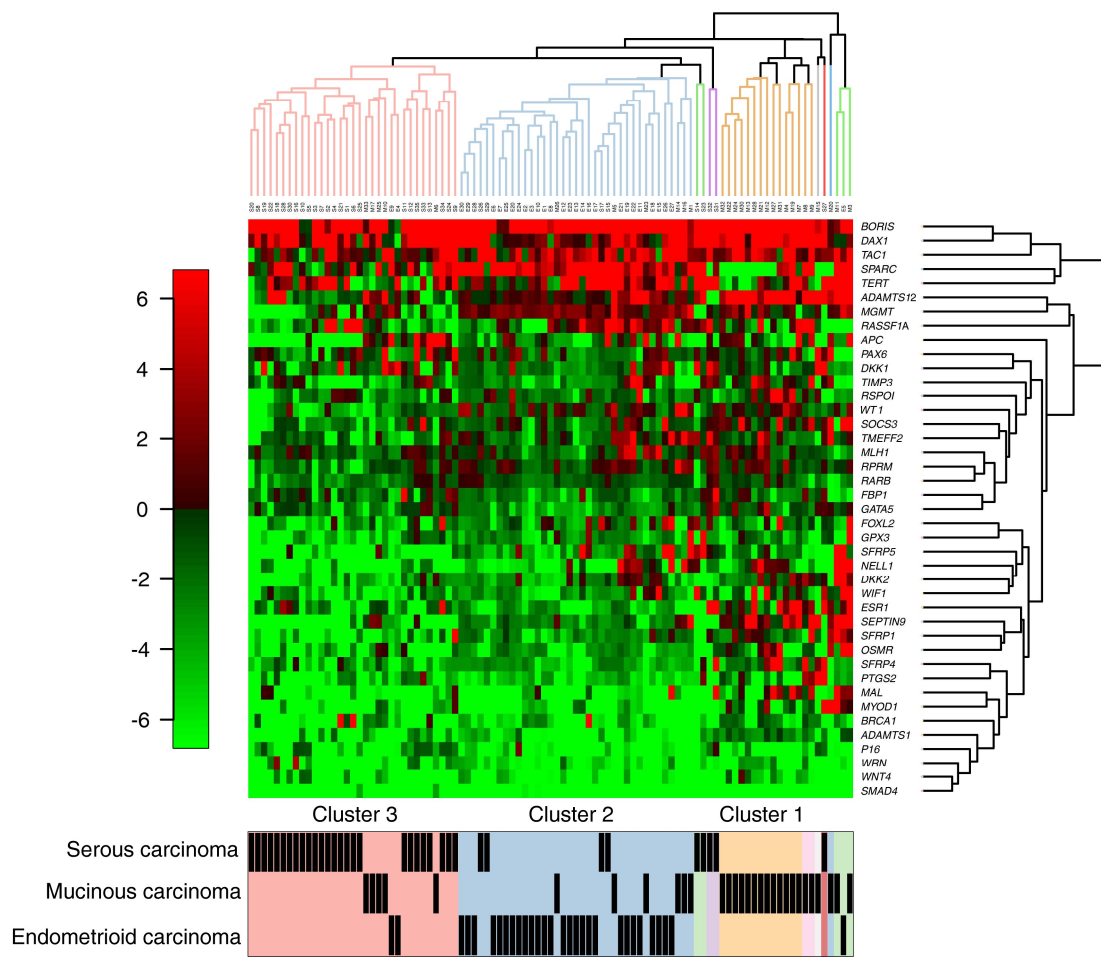

Figure S1. Heatmap\_EMS.

Supplement: Supplementary file 1 [file ijms-19-01559-s001.pdf]
